# Muscle mass and body composition in Japanese children measured by bioelectrical impedance analysis

**DOI:** 10.1038/s41598-025-22830-5

**Published:** 2025-11-10

**Authors:** Hideki Nakayama, Mototsugu Shimokawa, Tamaki Ueda, Maiko Noguchi, Shunichiro Toya, Wakako Kato, Utako Oba, Satoshi Honjo

**Affiliations:** 1 Division of Pediatrics, NHO Kyushu Cancer Center, 3-1-1, Notame, Minami-ku, Fukuoka City, Fukuoka 811-1395 Japan; 2 Clinical research center, NHO Kyushu Cancer Center, 3-1-1, Notame, Minami-ku, Fukuoka City, Fukuoka 811-1395 Japan; 3 Department of Pediatrics, Kyushu University, 3-1-1, Maidashi, Higashi-ku, Fukuoka City, Fukuoka 812-8582 Japan; 4 International University of Health and Welfare School of Health Sciences at Fukuoka, 137-1 Enokizu, Okawa City, Fukuoka 831-8501 Japan; 5 Department of Clinical Research, NHO Fukuoka National Hospital, 4-39-1, Yakatabaru, Minami-ku, Fukuoka City, Fukuoka 811-1394 Japan; 6 Yanagawa Institute for Developmental Disabilities, 218-1, Tano-machi, Mitsuhashimachi, Yanagawa City, Fukuoka 832-0813 Japan

**Keywords:** Adolescent, Bioelectrical impedance, Body composition, Body fat percentage (BFP), Children, Skeletal muscle mass index (SMI), Health care, Medical research, Risk factors

## Abstract

Age- and sex-specific reference values for muscle mass and body composition are essential for evaluating childhood cancer survivors at risk of muscle loss and abnormal body composition. To establish standard values for Japanese children, we conducted a cross-sectional study of healthy children aged 6 to 17 years. From April to June 2023, height, weight, and body composition were measured via bioelectrical impedance analysis (Tanita MC 780 A-N) in 9,202 students at 16 schools in Fukuoka City and nearby areas in Japan. Boys had significantly higher lean mass index (LMI) than girls at all ages (*p* < 0.001). In boys, LMI and skeletal muscle mass index (SMI) showed strong positive correlations with age (γ = 0.748, γ = 0.806), whereas girls exhibited moderate correlations (γ = 0.482, γ = 0.622). Although no sex difference in SMI was observed at age 6, boys surpassed girls thereafter. Trunk LMI increased linearly with age in boys (γ = 0.904), while in girls it plateaued around age 13 (γ = 0.819). Upper limb LMI peaked in girls around 14 years (γ = 0.771), with strong growth seen in boys (γ = 0.824). Lower limb LMI correlated strongly with age in both sexes (boys γ = 0.909, girls γ = 0.868). Girls had consistently higher fat mass index (FMI) and body fat percentage (BFP) across all ages (*p* < 0.001). Boys showed little correlation between FMI/BFP and age (γ = 0.117, γ = 0.004), whereas girls exhibited weak positive correlations (γ = 0.544, γ = 0.595). In healthy Japanese children aged 6 to 17 years, boys show a progressive increase in lean body mass and muscle mass, whereas girls demonstrate an age-related increase in fat mass. These normative data would offer a useful reference for monitoring body composition in children, particularly in targeted interventions for childhood cancer survivors.

**Supplementary Information:**

The online version contains supplementary material available at 10.1038/s41598-025-22830-5.

## Introduction

Body mass index (BMI) is commonly used to assess obesity or thinness in adults, but it is unsuitable for children due to individual differences in growth and rapid changes in height and weight^1,2^. And BMI doesn’t distinguish between fat and muscle mass, making it an incomplete measure. Therefore, evaluating children’s weight status requires supplementing BMI with growth charts and body composition data^3^. Body composition refers to the division of the body into fat and non-fat components, with the non-fat portion further divided into bone and muscle. Assessing body composition allows for the evaluation of muscle mass and fat mass, making it possible to monitor the effects of disease and treatment^4^. Recently, body composition analysis has attention across various fields, particularly in evaluating muscle loss^5^. The concept and application of sarcopenia have become widespread in the assessment of physical function in the elderly^6,7^. Moreover, abnormalities in body composition have also been reported in children with a wide range of pediatric conditions, including hematological and neoplastic diseases, chronic inflammatory liver disease, fatty liver, endocrine disorders, neuromuscular diseases, and childhood obesity^8–12^. Establishing standard reference values for body composition during childhood is essential for managing and monitoring children with diverse clinical conditions^9,10,13^. Although the cross-sectional areas of the psoas muscles quantified on CT or MRI is an established method for evaluating skeletal muscle mass^14–16^, large-scale studies could be conducted with bioelectrical impedance analysis (BIA)^17–19^. BIA estimates body composition by measuring the impedance of a weak electrical current passed through the body and applying regression equations^20^. While BIA is generally less accurate than dual-energy X-ray absorptiometry (DXA), it offers a convenient, non-invasive means to assess body composition and is suitable for large-scale surveys involving thousands of participants^17–19^. However, their overseas findings cannot be directly applied to Japanese children due to differences in race, ethnicity, and lifestyle factors.

At NHO Kyushu Cancer Center, sarcopenia was observed in 11% of adult survivors of pediatric leukemia and lymphoma, around 20 years after completing treatment^21^. Goodenough et al. reported that muscle wasting and weakness were associated with other late effects, beginning at diagnosis and progressing throughout treatment in childhood cancers^22^. Therefore, baseline data on body composition during childhood are essential for evaluating muscle mass and long-term outcomes in survivors of pediatric cancer. Due to the lack of large-scale data on body composition during the growth period in Japan, we conducted a cross-sectional survey of children aged 6 to 17 years using a BIA-based body composition analyzer as a more feasible alternative, since it is practically difficult to measure the body composition of a large number of school children using the DXA method.

## Methods

We hypothesized that by measuring groups of more than 200 children of each age and sex, without any special selection criteria, we would be able to calculate reliable averages and standard deviations for each group. Assuming a parental consent rate of 50%, we planned to survey 24 groups of 1st to 12th-grade boys and girls, with approximately 400 students in each group, totaling over 9600 participants. In 2023, we enrolled 10,853 children from seven elementary schools, five junior high schools, and four high schools in Fukuoka City and surrounding areas. Children who were deemed unable to participate due to health or physical conditions were excluded. Between April 10 and June 12, 2023, body composition measurements were conducted for 9,202 children (84.8%; 4,863 boys and 4,339 girls) whose guardians had provided informed consent to participate in the study.

### Survey

An explanatory document regarding the purpose and methods of the survey was distributed to students at elementary schools, junior high schools, high schools that agreed to cooperate with our study. Body composition was measured using an eight-electrode body composition device (MC-780 A-N; Tanita Corporation, Tokyo, Japan). The participants removed their socks and shoes, and stepped on the body composition device before they ate lunch.

### Ethical considerations

This study was reviewed by the Clinical Research Committee of Kyushu Cancer Center and approved by the Ethics Committee (No. 2022-31), in accordance with the Declaration of Helsinki^23^ and the Ethical Guidelines for Medical and Biological Research Involving Human Subjects established by the Ministry of Education, Culture, Sports, Science and Technology and the Ministry of Health, Labour and Welfare of Japan^24^. Informed consents were obtained from all participants and/or their legal guardians.

### Calculations

The body mass index (BMI) was calculated as body weight (kg) divided by height squared (m^2^). BMI was used to evaluate obesity and thinness. The fat mass index (FMI) was calculated as body fat mass (kg) divided by height squared (m^2^). The lean mass index (LMI) was calculated as lean mass (kg) divided by height squared (m^2^). The skeletal muscle mass index (SMI) was calculated as the appendicular skeletal muscle mass of the limbs (kg) divided by height squared (m^2^). The body fat percentage (BFP) was calculated as follows: [body fat mass (kg) / body weight (kg)] × 100. The BFP referred to the percentage of fat mass comprising the body weight, and its evaluation criteria differ depending on the physical characteristics of male and female individuals. LMI of trunks was calculated as lean mass of trunks (kg) divided by height squared (m^2^). LMI of upper limbs and lower limbs are calculated as lean mass of upper limbs (kg) and lower limbs (kg) divided each by height squared (m^2^).

### Statistical analysis

The mean and standard deviation (SD) of each body composition measurement were calculated by age and sex. The normality of the age-sex distribution of the measurements was assessed using the Shapiro–Wilk test. A p-value ≥ 0.05 was considered indicative of a normal distribution. A comparison of boys and girls was performed using the Mann-Whitney U test. The calendar age on the day of measurement was calculated based on the month of birth and the date of measurement, and the correlation between the calendar age and measurement values was evaluated using Pearson’s correlation coefficient. EZR version 1.52 was used for statistical analyses^25^.

### Protection of personal information

During this study, the names and student identification numbers of the participants were replaced with research identification numbers to protect their personal information, and all data were anonymized. When conducting the survey, those involved in obtaining the measurements were careful to protect the personal information of the participants.

## Results

The chronological age, height, weight, BMI, and body composition parameters of the children are presented by age in Tables 1 and 2. With respect to height, boys were significantly taller than girls at age 7 (Mann-Whitney U-test, *p* = 0.02) and from age 12 onwards (*p* < 0.001), whereas girls were significantly taller than boys at age 11 (*p* = 0.019). Regarding weight, boys weighed significantly more than girls at ages 7 (*p* = 0.037), 8 (*p* = 0.010), and from age 13 onwards (*p* < 0.001). For BMI, boys had significantly higher values than girls at age 8 (*p* = 0.07), while girls had significantly higher BMI at ages 13 (*p* = 0.004), 14 (*p* = 0.001), and 16 (*p* = 0.032). No significant sex differences in BMI were observed at other ages.

**Table 1:** Body composition parameters in Japanese children aged 6–11 years.

Age		Years	6				7				8				9				10				11			
Sex	Parameter	Units	Median	Q1	Q3	*p*	Median	Q1	Q3	*p*	Median	Q1	Q3	*p*	Median	Q1	Q3	*p*	Median	Q1	Q3	*p*	Median	Q1	Q3	*p*
Male	Number		207				200				216				237				225				241			
	CA	years	6.6	6.4	6.8	N.D	7.7	7.4	7.8	N.D	8.6	8.3	8.8	N.D	9.7	9.3	9.9	N.D	10.6	10.4	10.8	N.D	11.7	11.4	11.8	N.D
	Height	cm	116.7	113.6	120.7	N.D	122.4	119.5	126.2	0.020	128.5	125.5	132.0	N.D	134.7	131.0	138.0	N.D	139.7	135.8	144.3	N.D	147.0	142.0	151.2	F
	Weight	kg	20.8	19.1	22.6	N.D	23.6	21.3	25.9	0.037	27.2	24.4	30.6	0.010	29.7	27.3	33.2	N.D	32.9	29.8	39.0	N.D	38.2	33.7	43.4	N.D
	BMI	kg/m^2^	15.1	14.5	16.1	N.D	15.5	14.7	16.5	N.D	16.3	15.3	17.8	0.007	16.5	15.5	17.6	N.D	16.8	15.7	18.9	N.D	17.8	16.5	19.7	N.D
	LMI	kg/m^2^	12.8	12.6	13.2	< 0.001	13.0	12.7	13.3	< 0.001	13.3	13.0	13.7	< 0.001	13.5	13.1	13.8	< 0.001	13.7	13.3	14.1	< 0.001	14.2	13.7	14.7	< 0.001
	SMI	kg/m^2^	5.3	5.0	5.6	N.D	5.5	5.2	5.8	< 0.001	5.8	5.5	6.1	< 0.001	5.9	5.7	6.2	< 0.001	6.2	5.9	6.5	< 0.001	6.6	6.2	7.1	< 0.001
	LMI-trunks	kg/m^2^	7.6	7.5	7.7	< 0.001	7.5	7.4	7.6	< 0.001	7.5	7.4	7.7	< 0.001	7.5	7.4	7.7	< 0.001	7.5	7.3	7.7	< 0.001	7.6	7.4	7.8	< 0.001
	LMI-upper	kg/m^2^	1.0	0.9	1.0	F	1.0	1.0	1.1	N.D	1.1	1.0	1.1	< 0.001	1.1	1.1	1.2	< 0.001	1.1	1.1	1.2	< 0.001	1.2	1.1	1.3	< 0.001
	LMI-lower	kg/m^2^	4.3	4.1	4.5	0.03	4.5	4.3	4.7	< 0.001	4.7	4.5	5.0	< 0.001	4.8	4.7	5.1	< 0.001	5.0	4.8	5.3	< 0.001	5.4	5.1	5.7	< 0.001
	FMI	kg/m^2^	1.7	1.4	2.3	F	1.9	1.4	2.5	F	2.2	1.7	3.4	F	2.3	1.6	3.3	F	2.5	1.7	4.0	F	2.7	2.0	4.1	F
	BFP	%	11.6	9.5	14.2	F	12.2	9.6	15.3	F	13.7	11.0	19.0	F	14.1	10.5	18.3	F	14.8	10.7	21.4	F	15.3	12.1	21.4	F
Female	Number		200				202				236				205				220				231			
	CA	years	6.6	6.3	6.9	N.D	7.6	7.3	7.8	N.D	8.6	8.3	8.8	N.D	9.6	9.3	9.8	N.D	10.6	10.3	10.8	N.D	11.7	11.4	11.8	N.D
	Height	cm	115.6	113.3	119.3	N.D	121.8	117.5	124.8	M	127.6	124.1	131.2	N.D	134.1	129.7	138.0	N.D	141.6	135.7	146.9	N.D	148.5	143.1	152.7	0.019
	Weight	kg	20.6	18.8	23.0	N.D	22.7	20.9	25.2	M	25.7	23.5	29.5	M	29.4	26.0	33.3	N.D	34.2	29.2	39.3	N.D	39.1	34.5	43.5	N.D
	BMI	kg/m^2^	15.3	14.5	16.3	N.D	15.3	14.5	16.3	N.D	15.8	14.6	17.7	M	16.4	15.2	17.8	N.D	17.0	15.4	18.7	N.D	17.5	16.3	19.4	N.D
	LMI	kg/m^2^	12.4	12.1	12.9	M	12.5	12.1	13.0	M	12.7	12.1	13.3	M	12.8	12.3	13.5	M	12.9	12.4	13.5	M	13.2	12.7	13.8	M
	SMI	kg/m^2^	5.2	5.0	5.5	N.D	5.3	5.0	5.6	M	5.4	5.1	5.8	M	5.6	5.3	5.9	M	5.7	5.4	6.1	M	5.9	5.5	6.2	M
	LMI-trunks	kg/m^2^	7.2	7.0	7.5	M	7.2	7.0	7.5	M	7.2	6.9	7.6	M	7.3	7.0	7.5	M	7.3	6.9	7.6	M	7.4	7.1	7.7	M
	LMI-upper	kg/m^2^	1.0	0.9	1.1	0.01	1.0	0.9	1.1	N.D	1.0	1.0	1.1	M	1.0	1.0	1.1	M	1.1	1.0	1.1	M	1.1	1.0	1.2	M
	LMI-lower	kg/m^2^	4.2	4.0	4.5	M	4.3	4.1	4.5	M	4.4	4.1	4.7	M	4.5	4.3	4.8	M	4.7	4.4	4.9	M	4.8	4.5	5.0	M
	FMI	kg/m^2^	2.1	1.7	2.8	< 0.001	2.3	1.7	2.8	< 0.001	2.5	1.8	3.6	0.03	2.7	2.1	3.8	< 0.001	3.1	2.2	4.3	< 0.001	3.5	2.7	4.5	< 0.001
	BFP	%	14.6	11.9	17.1	< 0.001	14.6	11.9	17.8	< 0.001	15.8	12.5	20.9	< 0.001	16.8	13.9	21.2	< 0.001	18.3	14.6	22.9	< 0.001	19.7	16.6	23.5	< 0.001

Q1, The first quartile (25 percentile) value; Q3, the third quartile (75 percentile) value.

The *P* value indicates the result of testing the difference between men and women using the Mann–Whitney test. CA, calendar age; N.D., no difference; F, female data is significantly higher than male data; BMI, body mass index; LMI, lean mass index; SMI, skeletal muscle mass index; FMI, fat mass index; BFP, body fat percentage; M, male data is significantly higher than female data.

The boxes of normally distributed data (*p* ≧ 0.05, Shapio-Wilk normality test) are shown in a lighter shade.

**Table 2:** Body composition parameters in Japanese children aged 12–17 years.

Age		Years	12				13				14				15				16				17			
Sex	Parameter	Units	Median	Q1	Q3	*p*	Median	Q1	Q3	*p*	Median	Q1	Q3	*p*	Median	Q1	Q3	*p*	Median	Q1	Q3	*p*	Median	Q1	Q3	*p*
Male	Number		363				304				355				870				861				784			
	CA	years	12.6	12.3	12.8	N.D	13.6	13.4	13.9	N.D	14.6	14.3	14.8	N.D	15.6	15.4	15.8	N.D	16.6	16.4	16.9	N.D	17.6	17.4	17.9	N.D
	Height	cm	154.4	148.4	159.4	N.D	162.0	157.3	167.0	0.020	165.8	162.4	169.6	N.D	168.5	164.8	172.9	N.D	170.1	166.6	174.0	N.D	170.9	166.8	174.6	0.019
	Weight	kg	43.8	37.8	50.1	N.D	48.7	44.5	53.7	0.037	52.8	47.7	58.6	0.010	56.1	51.4	62.1	N.D	57.8	52.7	63.9	N.D	59.9	54.8	65.9	N.D
	BMI	kg/m^2^	18.0	16.7	20.3	N.D	18.4	17.3	20.1	N.D	19.0	17.6	21.1	0.007	19.6	18.3	21.5	N.D	19.9	18.5	21.7	N.D	20.4	19.0	22.1	N.D
	LMI	kg/m^2^	14.5	14.0	15.2	< 0.001	15.0	14.6	15.6	< 0.001	15.4	14.8	16.2	< 0.001	15.9	15.2	16.8	< 0.001	16.3	15.5	17.2	< 0.001	16.6	15.7	17.7	< 0.001
	SMI	kg/m^2^	6.8	6.4	7.2	N.D	7.1	6.8	7.5	< 0.001	7.4	7.0	7.8	< 0.001	7.7	7.3	8.2	< 0.001	7.9	7.5	8.4	< 0.001	8.1	7.6	8.7	< 0.001
	LMI-trunks	kg/m^2^	7.7	7.5	8.1	< 0.001	7.9	7.7	8.2	< 0.001	8.1	7.8	8.4	< 0.001	8.2	7.9	8.7	< 0.001	8.4	7.9	8.8	< 0.001	8.5	8.0	9.1	< 0.001
	LMI-upper	kg/m^2^	1.3	1.2	1.4	F	1.4	1.3	1.5	N.D	1.5	1.4	1.5	< 0.001	1.5	1.4	1.6	< 0.001	1.5	1.4	1.7	< 0.001	1.6	1.4	1.7	< 0.001
	LMI-lower	kg/m^2^	5.5	5.2	5.8	0.03	5.7	5.4	6.0	< 0.001	5.9	5.6	6.2	< 0.001	6.2	5.8	6.5	< 0.001	6.4	6.0	6.8	< 0.001	6.5	6.1	7.0	< 0.001
	FMI	kg/m^2^	2.6	1.9	4.2	F	2.4	1.9	3.6	F	2.7	1.9	3.8	F	2.8	2.1	4.0	F	2.7	2.0	3.6	F	2.9	2.2	3.9	F
	BFP	%	14.3	11.2	20.5	F	13.2	10.7	17.9	F	13.8	10.8	18.5	F	14.4	11.2	18.5	F	13.5	10.9	17.2	F	14.3	11.5	17.7	F
Female	number		343				331				345				722				624				680			
	CA	years	12.6	12.3	12.8	N.D	13.5	13.3	13.8	N.D	14.6	14.3	14.8	N.D	15.6	15.3	15.8	N.D	16.6	16.3	16.8	N.D	17.6	17.4	17.9	N.D
	Height	cm	152.0	147.0	155.4	N.D	155.1	151.7	158.6	M	156.0	152.4	160.0	N.D	157.2	153.8	160.4	N.D	157.8	154.2	161.9	N.D	158.1	154.8	161.8	M
	Weight	kg	42.7	38.3	47.5	N.D	45.6	42.0	50.6	M	48.7	44.8	52.8	M	49.5	45.1	54.2	N.D	50.5	46.6	55.0	N.D	50.8	46.8	55.4	N.D
	BMI	kg/m^2^	18.4	17.1	20.1	N.D	19.0	17.8	20.6	N.D	19.6	18.5	21.4	M	20.0	18.5	21.6	N.D	20.3	18.9	21.8	N.D	20.2	19.1	21.7	N.D
	LMI	kg/m^2^	13.4	12.8	14.0	M	13.5	13.0	14.1	M	13.7	13.2	14.2	M	13.8	13.2	14.4	M	14.0	13.3	14.6	M	13.9	13.3	14.6	M
	SMI	kg/m^2^	5.9	5.5	6.3	N.D	5.9	5.6	6.3	M	6.2	5.8	6.5	M	6.4	6.0	6.7	M	6.5	6.1	6.9	M	6.5	6.2	6.9	M
	LMI-trunks	kg/m^2^	7.5	7.3	7.8	M	7.6	7.4	7.8	M	7.5	7.3	7.7	M	7.4	7.2	7.7	M	7.4	7.1	7.7	M	7.4	7.1	7.7	M
	LMI-upper	kg/m^2^	1.2	1.1	1.3	0.01	1.2	1.1	1.3	N.D	1.2	1.1	1.3	M	1.2	1.1	1.3	M	1.2	1.1	1.3	M	1.2	1.1	1.3	M
	LMI-lower	kg/m^2^	4.7	4.4	5.0	M	4.8	4.4	5.1	M	5.0	4.7	5.3	M	5.1	4.9	5.4	M	5.3	5.0	5.6	M	5.4	5.1	5.6	M
	FMI	kg/m^2^	4.2	3.3	5.4	< 0.001	4.6	3.7	5.7	< 0.001	5.2	4.3	6.3	0.03	5.3	4.4	6.4	< 0.001	5.5	4.5	6.4	< 0.001	5.5	4.5	6.4	< 0.001
	BFP	%	22.7	19.1	27.1	< 0.001	24.1	20.9	28.1	< 0.001	26.1	22.9	30.0	< 0.001	26.7	23.6	30.1	< 0.001	27.0	23.6	30.1	< 0.001	27.1	23.6	30.1	< 0.001

Q1, The first quartile (25 percentile) value; Q3, the third quartile (75 percentile) value.

The *P* value indicates the result of testing the difference between men and women using the Mann–Whitney test. CA, calendar age; N.D., no difference; F, female data is significantly higher than male data; BMI, body mass index; LMI, lean mass index; SMI, skeletal muscle mass index; FMI, fat mass index; BFP, body fat percentage; M, male data is significantly higher than female data.

The boxes of normally distributed data (*p* ≧ 0.05, Shapio-Wilk normality test) are shown in a lighter shade.

### LMI and SMI

**Fig. 1:**
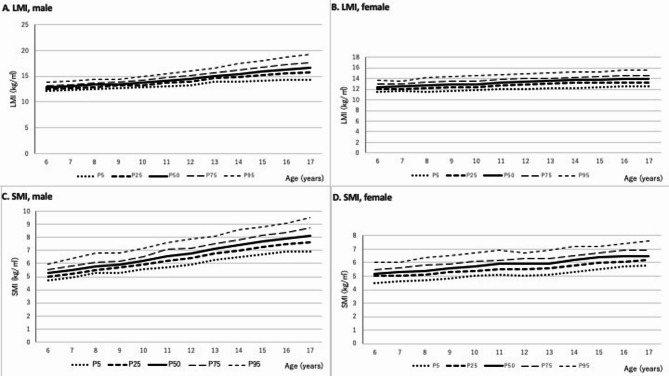
Age-specific and sex-specific distributions of lean mass index (LMI) and skeletal muscle mass index (SMI) in children aged 6 to 17. The bottom line in each figure indicates the 5th percentile, the second line indicates the 25th percentile, the third line indicates the 50th percentile, the fourth line indicates the 75th percentile, and the top line indicates the 95th percentile, respectively. (**A**) LMI of male, (**B**) LMI of female, (**C**) SMI of male, (**D**) SMI of female.

The median, first quartile and third quartile values of LMI or SMI by gender for ages are shown in Tables 1 and 2. Boys exhibited significantly higher LMI values than girls at all ages from 6 to 17 years (*p* < 0.001). At age 6, there was no statistically significant difference in SMI between boys (median: 5.3 kg/m²) and girls (5.2 kg/m²). However, from age 7 onward, boys consistently had significantly higher SMI than girls (*p* < 0.001). Figure 1 shows the distributions of LMI and SMI by age, including the 5th, 25th, 50th, 75th, and 95th percentiles. A strong positive correlation between LMI and age was observed in boys (γ = 0.748; 95% CI: 0.735–0.760; Fig. 1-A), while a weaker correlation was seen in girls (γ = 0.482; 95% CI: 0.459–0.505; Fig. 1-B). SMI increased from a median of 5.2 ± 0.41 kg/m² at age 6 to a mean of 8.1 ± 0.79 kg/m² at age 17 in boys, and from 5.2 ± 0.46 kg/m² to 6.5 ± 0.53 kg/m² in girls. A positive association with age was observed in both sexes (boys: γ = 0.806; 95% CI: 0.796–0.815; girls: γ = 0.622; 95% CI: 0.603–0.640; Fig. 1C and D).

### LMI by body parts

**Fig. 2:**
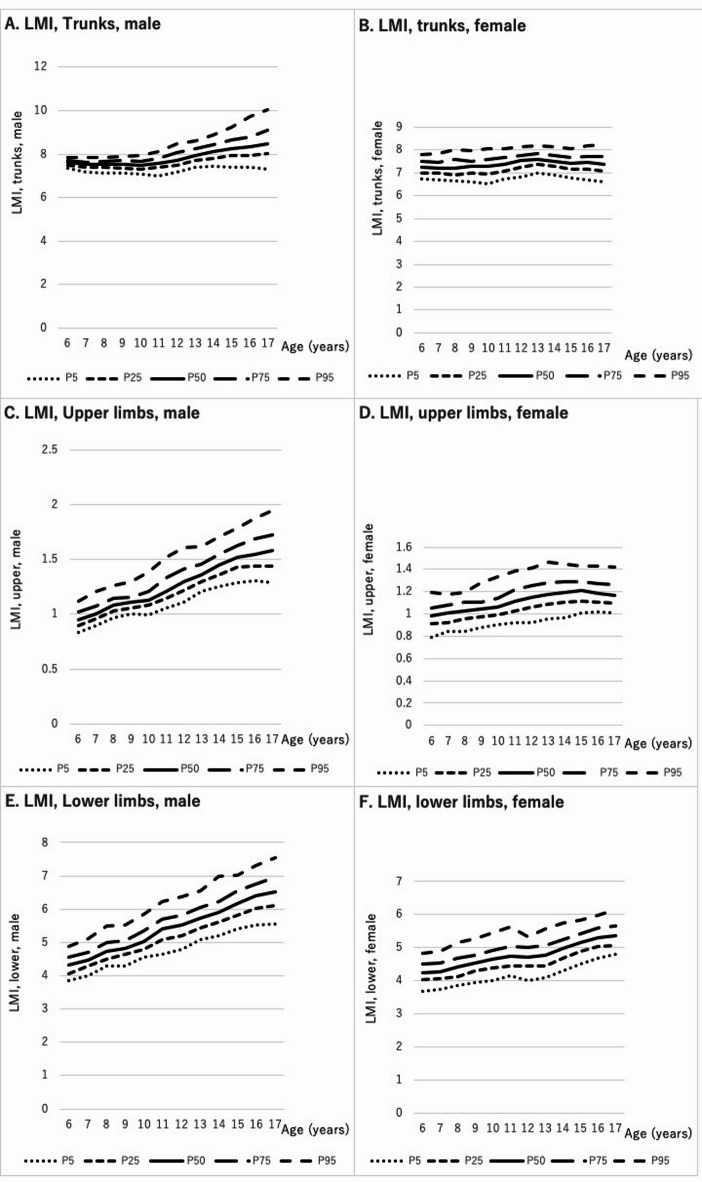
Age-specific and sex-specific distributions of lean mass index (LMI) according to body parts in children aged 6–17 years. The bottom line in each figure indicates the 5th percentile, the second line indicates the 25th percentile, the third line indicates 50th percentile, the fourth line indicates the 75th percentile, and the top line indicates the 95th percentile, respectively. (**A**) Trunks of male, (**B**) Trunks of female, (**C**) Upper limbs of male, (**D**) Upper limbs of female, (**E**) Lower limbs of male, (**F**) Lower limbs of female.

Trunk LMI was significantly higher in boys than in girls at all ages (*p* < 0.001). Trunk LMI increased steadily with age in boys (γ = 0.904; 95% CI: 0.899–0.909; Fig. 2-A), while in girls, it peaked at 7.6 kg/m² at age 13 and slightly declined to 7.4 kg/m² by age 17 (Fig. 2-B), though a strong correlation with age remained (γ = 0.819; 95% CI: 0.809–0.828).

Upper limb LMI was significantly higher in girls (0.99 kg/m²) than in boys (0.96 kg/m²) only at age 6 (*p* = 0.012). No significant difference was observed at age 7. From age 8 onward, boys showed significantly higher upper limb LMI (*p* < 0.001). A strong correlation with age was found in boys (γ = 0.824; 95% CI: 0.814–0.832; Fig. 2-C) and in girls (γ = 0.771; 95% CI: 0.758–0.783; Fig. 2-D), although the increase plateaued around age 15 in girls.

Lower limb LMI was consistently higher in boys than girls across all ages (*p* = 0.030 at age 6; *p* < 0.001 at all other ages). A strong positive correlation with age was observed in both sexes (boys: γ = 0.909; 95% CI: 0.904–0.914; girls: γ = 0.868; 95% CI: 0.860–0.875).

### FMI and BFP

**Fig. 3:**
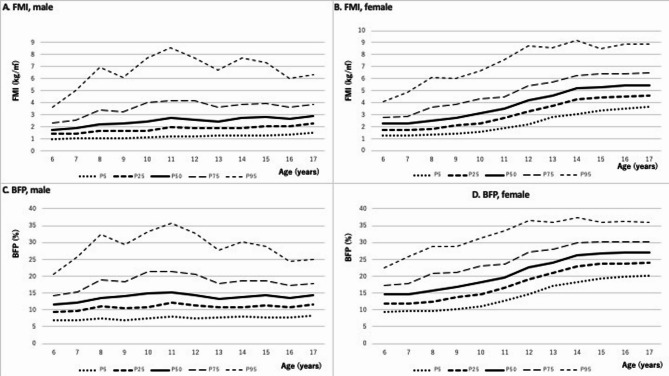
Age-specific and sex-specific distributions of fat mass index (FMI) and body fat percentage (BFP) in children aged 6–17 years. The bottom line in each figure indicates the 5th percentile, the second line indicates the 25th percentile, the third line indicates the 50th percentile, the fourth line indicates the 75th percentile, and the top line indicates the 95th percentile, respectively. (**A**) FMI of male, (**B**) FMI of female, (**C**) BFP of male, (**D**) BFP of female.

Girls exhibited significantly higher FMI than boys at all ages (*p* = 0.027 at age 8; *p* < 0.001 at all other ages). Boys’ FMI showed no significant correlation with age (γ = 0.117; 95% CI: 0.0895–0.145; Fig. 3A), peaking at 3.47 kg/m² at age 11. In contrast, girls’ FMI showed a weak positive correlation with age (γ = 0.544; 95% CI: 0.523–0.565; Fig. 3B).

Girls also had significantly higher BFP than boys across all ages (6–17 years; *p* < 0.001). In boys, BFP remained relatively stable, ranging from 12.47% at age 6 to 17.54% at age 11 (γ = 0.004; 95% CI: −0.0238 to 0.0325; Fig. 3C). Girls’ BFP increased gradually from 15.11% at age 6 to 27.40% at age 17 (γ = 0.595; 95% CI: 0.575–0.613; Fig. 3D).

## Discussion

This study enrolled over 9,000 participants, with more than 200 per age and gender from ages 6–11, over 300 in junior high, and 600–800 in high school. Larger samples in older age groups accounted for increased variability, ensuring statistical reliability. The study clarified sex- and age-specific trends in body composition among Japanese children aged 6 to 17, focusing on qualitative measures such as LMI, SMI, FMI, and BFP. These indicators provide a more nuanced understanding of growth and physical development than height and weight alone, with implications for health management and exercise programming in youth^1–4^.

Boys consistently had higher LMI and SMI, particularly after age 13, likely due to the anabolic effects of androgens, which enhance muscle protein synthesis^26^. SMI values at age 17 in boys (8.1 kg/m²) and girls (6.5 kg/m²) approached average levels observed in Japanese adults aged 60–69^27^. Conversely, estrogen promotes fat accumulation in girls, leading to a more gradual increase in muscle indices^28^. Regionally, boys had higher LMI in all body areas. Among girls, trunk muscle mass peaked during early adolescence and then plateaued^29^. Higher upper limb LMI in boys may be attributed to more frequent participation in outdoor or high-intensity sports activities^30^.

Girls had consistently higher FMI and BFP than boys, consistent with normal pubertal development and estrogen effects^31^. However, excessive fat gain can increase metabolic risk and insulin resistance, emphasizing the need for early interventions through balanced nutrition and physical activity^32^.

Compared to the 2000 School Health Statistical Survey^33^, the height and weight data in this study reflect national averages for Japanese children. Nevertheless, as the study excluded participants from non-Japanese backgrounds, findings should not be generalized to international populations, especially given Japan’s growing immigrant demographic. Large-scale BIA studies in other countries, including China^17^, Germany^18^, and Poland^19^, show that Japanese children tend to be shorter and lighter than their European peers. Additionally, Japanese children often have lower FFMI but may have higher FMI, especially in girls^18,19^. These comparisons support the importance of developing localized, age- and sex-specific reference values.

Less than 2% of participants had SMI below − 2 SD (data not shown), while a prior study of Japanese university students reported 8% of males and 3% of females with low SMI, with a sarcopenia prevalence of 1% in both sexes^35^. Currently, no simple and standardized method exists for assessing motor function in children, highlighting the need for new diagnostic tools for pediatric sarcopenia^36^.

With survival rates for pediatric cancer now exceeding 80%^37^, attention to long-term effects is essential^38,39^. Sarcopenia is one such late effect, especially in children treated with hematopoietic stem cell transplantation^21,22,40^, acute lymphoblastic leukemia, or solid tumors^11–15,21^. In the Netherlands, 4.4% of adult survivors of childhood cancer had sarcopenia^13^. Early detection and intervention through nutrition and exercise may be warranted, even if baseline muscle mass assessments were not conducted before treatment.

Due to the need for subjects to stand still for 15 s during BIA, alternative methods are necessary for children who cannot maintain this posture^16^. Devices capable of measuring muscle mass in the supine position are also needed.

This study had some limitations. This was a cross-sectional study conducted within a single year, with no longitudinal follow-up. Participants were limited to the Fukuoka City area, and fewer younger children were included compared to older ones. Additionally, data on physical activity, diet, and other potential confounders were not collected. COVID-19–related lifestyle restrictions over the previous 3–4 years may have also affected muscle development [41, 42]. Future studies should expand geographically and temporally to validate these findings.

## Conclusion

This study revealed clear age- and sex-related differences in body composition among healthy Japanese children aged 6–17. Boys showed a progressive increase in lean and muscle mass, while girls exhibited a gradual increase in fat mass. These normative data can serve as valuable reference points for monitoring body composition and designing targeted interventions, especially in populations such as childhood cancer survivors.

## Supplementary Information

Below is the link to the electronic supplementary material.

Supplementary Material 1

Supplementary Material 2

## Data Availability

The datasets generated and/or analyzed during this study were not publicly available, as participants and/or guardians did not provide consent for the general release of the raw data. However, they were available from the corresponding author upon reasonable request.
